# Genomic and phenomic predictions help capture low-effect alleles promoting seed germination in oilseed rape in addition to QTL analyses

**DOI:** 10.1007/s00122-024-04659-0

**Published:** 2024-06-11

**Authors:** Marianne Laurençon, Julie Legrix, Marie-Hélène Wagner, Didier Demilly, Cécile Baron, Sophie Rolland, Sylvie Ducournau, Anne Laperche, Nathalie Nesi

**Affiliations:** 1 Institute of Genetics, Environment and Plant Protection (IGEPP), INRAE – Institut Agro Rennes-Angers – Université de Rennes, 35650 Le Rheu, France; 2 Groupe d’Etude et de Contrôle des Variétés Et des Semences (GEVES), 49070 Beaucouzé, France

## Abstract

**Key message:**

Phenomic prediction implemented on a large diversity set can efficiently predict seed germination, capture low-effect favorable alleles that are not revealed by GWAS and identify promising genetic resources.

**Abstract:**

Oilseed rape faces many challenges, especially at the beginning of its developmental cycle. Achieving rapid and uniform seed germination could help to ensure a successful establishment and therefore enabling the crop to compete with weeds and tolerate stresses during the earliest developmental stages. The polygenic nature of seed germination was highlighted in several studies, and more knowledge is needed about low- to moderate-effect underlying loci in order to enhance seed germination effectively by improving the genetic background and incorporating favorable alleles. A total of 17 QTL were detected for seed germination-related traits, for which the favorable alleles often corresponded to the most frequent alleles in the panel. Genomic and phenomic predictions methods provided moderate-to-high predictive abilities, demonstrating the ability to capture small additive and non-additive effects for seed germination. This study also showed that phenomic prediction estimated phenotypic values closer to phenotypic values than GEBV. Finally, as the predictive ability of phenomic prediction was less influenced by the genetic structure of the panel, it is worth using this prediction method to characterize genetic resources, particularly with a view to design prebreeding populations.

**Supplementary Information:**

The online version contains supplementary material available at 10.1007/s00122-024-04659-0.

## Introduction

Oilseed rape (*Brassica napus* L.) is the leading oilseed crop in Europe (FAO 2023), but it faces numerous constraints, especially in a context of climate change and reduced chemical inputs. One of the keys to overcome these challenges would be to ensure successful plant establishment, enabling the crop to compete with weeds and tolerate stresses during the earliest developmental stages in order to guarantee a high-yield potential (Nelson et al. 2022). Indeed, oilseed rape establishment is affected by a wide range of biotic and abiotic stresses, such as seed- and soil-borne pathogens causing damping-off, pest attacks reducing young plantlet biomass (slugs and flea beetles), and weed competition as well as environmental limiting factors, especially water (lack or excess) and extreme temperatures that can occur after sowing or during plant emergence (Haj Sghaier et al. 2022). These stresses can dramatically reduce plant density and biomass thus, ultimately affecting yield. Successful plant establishment results from a combination of developmental processes of which germination is the first step (Rajjou et al. 2012). As a matter of fact, rapid and highly efficient germination combined to uniform plant emergence strongly increase the likelihood of plant establishment, which is expected especially under adverse environmental conditions.

Seed germination is a complex trait subject to genetic and environmental controls. The environmental effect is generally referred to the so-called ‘seed-lot’ effect that encompasses the conditions during seed development on the mother plant as well as the post-harvest conditions (Rajjou et al. 2012; Finch-Savage and Bassel 2016). In addition, the cultural practices also lead to variation in germination ability when considering factors such as the sowing date, seedbed preparation, intercropping, and choice of previous crop (Elliott et al. 2007; Lamichhane et al. 2018). Several studies have reported the polygenic nature of seed germination, which is under the control of numerous loci with moderate-effect each (Hatzig et al. 2015; Nguyen et al. 2018; Gad et al. 2021; Luo et al. 2021).

Modeling low additive effects distributed along the genome can be addressed through the recent development of prediction methods. The benchmark article of Meuwissen et al. (2001) conceptualized the prediction of complex traits using genotypic data, called genomic prediction (GP). GP combine phenotypic data and high-density molecular markers obtained on genotypes from a training population, to predict the genomic estimated breeding values (GEBV) of non-phenotyped genotypes from a testing population. Combined with GWAS approach, GP can accelerate genetic gain in breeding (Hickey et al. 2017; Araus et al. 2018). Other emerging methods have focused on using endophenotypes as trait predictors to complement or even to replace molecular data. This enables to improve not only the modeling of additive effects, but also of non-additive and epistatic effects (Mackay et al. 2009; Patti et al. 2012; Ritchie et al. 2015). Multiple studies integrated transcriptomic and metabolomic data, as endophenotypes, into predictive models, either in combination with genomic data or not (Westhues et al. 2017; Schrag et al. 2018; Knoch et al. 2021). These integrations complemented the information provided by molecular markers and have demonstrated the ability to better capture small genetic effects. Rincent and coworkers (2018) proposed a low-cost and high-throughput method as an alternative to the use of -omics data, based on near-infrared spectroscopy (NIRS), called phenomic prediction (PP).

Up to now, most of the studies reporting the use of GP and PP models have focused on traits related to seed yield, seed quality, or plant phenology mainly in cereal crops (Heffner et al. 2011; Albrecht et al. 2011; Rincent et al. 2018; Voss-Fels et al. 2019; Lane et al. 2020; Robert et al. 2022), with additional examples in other crops such as rapeseed (Würschum et al. 2014; Werner et al. 2018; Knoch et al. 2021) or other species such as trees (Resende et al. 2012; Muranty et al. 2015; Isik et al. 2016; Rincent et al. 2018; Brault et al. 2022). Considering seed germination phenotype, a first study reports the interest of GP to decipher the polygenic effect of seed germination capacity in barley (Rooney et al. 2022), with moderate-to-high predictive abilities.

As seed germination has rarely been considered as a selection trait in the past, it is very likely that certain genes and alleles of interest are present in both recent and ancient germplasms. Therefore, we investigate the genetic determinism of seed germination-related traits in rapeseed and the predictive ability of GP and PP models for these traits in a broad genetic pool including ancient and recent germplasm of winter, semi-winter, and spring type. The combination of different germplasms provided potential access to favorable low-effect polygenes. Our goal was to investigate whether GP and PP were promising methods to improve germination capacity in addition to GWAS. Our results led to (i) identify relationships between germination-related traits, (ii) decipher the genetic control of these traits, and (iii) compare multiple models of GP and PP, integrating one or multiple predictors, to evaluate the predictive abilities for seed germination-related traits.

## Material and methods

### Plant material and genotyping data

The diversity set consisted of 223 genetically diverse *B. napus* inbred lines, including 127 winter oilseed rape (WOSR), 81 spring oilseed rape (SOSR), 13 winter fodder (WFR), and two swedes (Online Resource 1). Seed lots were all produced in the field by open pollination during the 2020/2021 season in Le Rheu, France. Each genotype was sown in a four-row plot. To avoid pollen mixing, the outer rows of each plot were discarded at harvest. All the genotypes were sown and harvested at the same time. Seeds were then stored under the same conditions. After harvest, seed lot was split intro sub-lots each dedicated to a specific analysis or experiment (i.e., seed germination monitoring, TSW measurements, and acquisitions of NIRS spectra) as described below. All the accessions were genotyped using the *Brassica* 60 K Infinium® SNP array (Illumina, Inc., *San Diego*, CA) (Clarke et al. 2016), and the data were visualized using GenomeStudio software (Illumina, Inc., *San Diego*, CA). A total of 33,151 SNPs were validated using thresholds of 5% for the minor allele frequency (MAF), 10% for the frequency of missing values, and 10% for the heterozygosity level. The missing SNP data were imputed using BEAGLE software following the method described in Browning et al. (2018). SNPs were physically anchored to the *B. napus* Darmor-*bzh* v10 reference genome (Rousseau-Gueutin et al. 2020).

### Acquisition of germination data

Seed germination dynamics were monitored using the high-throughput phenotyping platform for germination at the National Seed Testing Station in Angers, France (PHENOTIC—Angers Seed Phenotyping Facility, Boureau 2020). Seeds were imbibed under controlled conditions at 20 °C, in the dark, during 96 h. The experimental design consisted of blocks of twenty-five seeds per genotype, repeated four times (Ducournau et al. 2004; Wagner et al. 2011). Imaging and analysis methods are described in Demilly et al. (2014), with pictures taken every 2 h over the experiment.

Germination parameters were monitored for each block of twenty-five seeds as following (Table 1): seed volume increase after 20 h of imbibition (VI, in %), final germination percentage after 96 h (GP, in %), first germination time (FG, in h) corresponding to the time when the first seed germinates, mean germination time (MGT, in h) corresponding to the mean delay to germinate for each seed lot, and radicle elongation speed (ES, in mm/h). Supplementary parameters were deduced from the germination dynamics, such as germination after 36 h of imbibition (GP36, in %), time to reach 20% of germination (T20, in h), time to reach 50% of germination (T50, in h), time to reach 80% of germination (T80, in h), uniformity (UNI) assessed as the difference between T80 and T20, and the area under the curve (AUC) that represents the germination rate as a function of the time from the initiation of the imbibition. AUC was estimated for each genotype using the values for the 100 seeds of the four blocks. Thousand seed weight (TSW, in g) was also measured for each seed lot by weighting seed samples after drying at 105 °C overnight.

**Table 1:** Variation of the germination-related traits assessed over a diversity germplasm of 223 rapeseed genotypes

Trait	Abb (unit)	Formula	Min	Max	Mean	SD	H^2^
Thousand seed weight	TSW (g)		2.550	6.250	4.358	0.623293	/
Seed volume increase after 20 h of imbibition	VI (%)	Volume_20 h_-Volume_0 h_	0.997	4.440	2.145	0.496137	0.40
Time of the 1st germ	FG (h)	$\frac{\sum {h}_{1\text{st germination}}}{\text{nrep}}$	22.00	58.00	30.25	4.805132	0.51
Time to 20% of germ	T20 (h)		28.50	64.00	52.26	7.252935	0.69
Time to 50% of germ	T50 (h)		36.00	79.67	51.93	8.855827	0.65
Time to 80% of germ	T80 (h)		43.20	96.00	63.55	11.15751	0.61
Mean germ. time	MGT (h)		38.12	75.43	52.26	6.849242	0.68
Uniformity	UNI (h)	T80-T20	11.53	50.00	24.04	7.472102	0.43
Area under the curve	AUC (%.h^−1^)	${\int }_{\text{h}2}^{\text{h}1}{f}_{\text{h}}{d}_{\text{h}}$	1444	5888	4077	972.7179	/
Germ. percentage at 36 h	GP36 (%)	$\frac{{\text{nb}}_{\text{germinated seeds at }36\text{h}}}{{\text{nb}}_{\text{total}}}*100$	0.00	52.00	15.49	10.35249	0.69
Germ. percentage at 96 h	GP (%)	$\frac{{\text{nb}}_{\text{germinated seeds at }96\text{h}}}{{\text{nb}}_{\text{total}}}*100$	46.67	100	49.46	10.91978	0.46
Radicle elongation speed	ES (mm.h^−1^)	$\frac{{\text{l}}_{\text{radicule at }16\text{h}}}{16\text{h}}$	0.080	0.209	0.149	0.026788	0.56

*Abb* trait abbreviation *Min, Max, Mean, and SD* minimum, maximum, mean, and standard deviation for fitted values *H*^*2*^ broad-sense heritability

### Phenotypic heritability

Broad-sense heritability (*H*^2^) was calculated for each phenotypic trait. Genotypic and error variance were extracted from the following linear mixed model:

$$Y_{i} = { }\mu + G_{i} + e_{i} ,$$

where *Y*_i_ is the trait value obtained for the genotype *i*, *G*_*i*_ corresponds to the random genetic effect for genotype i, and e_i_ is the residual effect. We assumed that *G*_*i*_ and *e*_*i*_ were independent, identically distributed and followed a normal distribution.

*H*^2^ was then calculated as follows: ${H}^{2}=\frac{{\sigma }_{G}^{2}}{{\sigma }_{G}^{2}+\frac{{\sigma }_{e}^{2}}{n}}$,where *σ*^2^_*G*_ is the genotypic variance, *σ*^2^_e_ is the error variance, and n is the number of repetitions.

### NIRS data

NIR spectra were collected on a sub-set of dry seed samples, that originated from the same seed lots as the sub-set used for the germination phenotyping experiments. A MPA FT-NIR spectrophotometer (Bruker Optic Inc., Germany) was used over the range of 4000–12,000 cm^−1^ with a 16 cm^−1^ optical resolution. NIRS was converted in nm in steps of 1 nm, so the final spectra range from 800 to 2781 nm. Three biological replicates were run per genotype, with each replicate being the average of 64 technical repetitions measured by the spectrophotometer. Due to a lack of seeds, only 210 genotypes were screened (Online Resource 1). NIR spectra were centered and scaled to reduce noise. Then, the first derivative of the Savitzky–Golay filter (Savitzky and Golay 1964) was calculated using the R package signal (Signal Developers 2014) to smooth the curve. The three replicates were then averaged to obtain a mean NIR spectrum per genotype.

### Exploratory analyses and correlations

To explore the relationship between the different seed germination traits, a principal component analysis (PCA) was performed using FactomineR package (Lê et al. 2008). The number of dimensions was chosen according to the percentage of explained variance. Then, a clustering was carried out, using the partitioning around medoids (PAM) method (Kaufman and Rousseeuw 1990). The number of clusters (*k*) was chosen using silhouette index and gap statistic from cluster R package (Maechler et al. 2022). Due to nonlinear relationships, Spearman correlations were calculated between traits.

### Population genetic structure and diversity

Genetic structure of the germplasm was unraveled using the first two components of a principal coordinates analysis (PCoA, Gower 1967) carried out using the SNP data. The PCoA is based on the genetic distances between genotypes estimated by the dissimilarity matrix (1-*K*). The genomic kinship (*K*) was estimated using Astle and Balding (2009) algorithm as following:

$$K= \frac{W{W}^{T}}{M}$$

With *W*, the matrix scaled on allelic frequencies with dimensions *N* × *M*, *N* is the number of genotypes and *M* is the number of molecular markers and *W*^*T*^ is the transposed *W* matrix.

Nucleotidic diversity (*π*) per chromosome was estimated using VCFtools (Danecek et al. 2011).

### Genome-wide association study

For the GWAS only, a kinship matrix was re-estimated for each chromosome tested as described by Rincent et al. (2014). The estimation of the kinship matrix (*K*) is similar to the methodology previously described, except that SNPs located on the chromosome under investigation were discarded. The pairwise linkage disequilibrium (LD) between SNPs (*r*^2^) was estimated per chromosome using PLINK software v.1.9 (Purcell et al. 2007). LD decay according to the physical distance between markers for each chromosome is represented in Online Resource 2.

GWAS was performed using FaST-LMM algorithm (Lippert et al. 2011). To reduce false associations (types I and II errors) due to population structure and kinship between genotypes, GWAS was performed with a mixed model that takes these two factors into account (Yu et al. 2006). Due to high average LD calculated between SNP pairs intra- and inter-chromosomes (mean LD = 0.51), the SimpleM approach (Gao et al. 2008, 2010) was used to reduce type I error (false-positive) by estimating the effective number of independent tests (M_eff_) based on composite LD for the GWAS. Markers were considered as significantly associated with a trait if the −log10 (*p*-value) exceeded a 5% threshold (using our data, the 5% threshold was 3.51). In addition, false discovery rate (FDR) adjusted *q*-values were calculated to reduce type I errors using the *q* value R package (Storey 2002). Markers with a *q*-value inferior to 0.2 were retained.

QTL confidence intervals were estimated based on the method proposed by Albert et al. (2016). Briefly, LD calculation was performed between 100,000 randomly chosen pairs of unlinked loci located on different chromosomes. The critical LD threshold was chosen as the 95th percentile of the LD distribution, which equaled 0.16 using our data. Then, local pairwise LD with markers located upstream and downstream the significant marker (on the same chromosome) was calculated in the same way as pairwise LD explained above. Confidence intervals were estimated for each significant marker as the interval between the first (upstream) and last (downstream) markers that presented a LD value higher than the LD threshold when compared to the significant marker.

Effects of each single QTL (*R*^2^ and allelic effect) were estimated at the peak marker that corresponded to the marker with the smallest p-value. Favorable alleles were identified for each QTL as the allele that improved the phenotype, that is to say increasing GP36, TSW, and VI values but decreasing MGT, T20, and T50 values. The number of favorable alleles across all QTL locations for each genotype and trait was calculated, ranging from 0 to the maximum number of QTL identified for the trait. An ANOVA and Tukey test at 5% probability level were performed to compare the effect of accumulating favorable alleles at QTL for each trait.

The genes under the identified QTL were obtained from the *B. napus* Darmor-*bzh* V10 annotation file (Rousseau-Gueutin et al. 2020). The function of candidate genes was obtained from TAIR (https://www.arabidopsis.org/).

### Genomic and phenomic predictions

Genomic heritability along NIR-based spectra was estimated for each wavelength from a statistical model considering a random polygenic effect. Genotypic and error variance were extracted from the following linear mixed model:

$${Y}_{ij}= \mu +{G}_{i}+{e}_{i},$$

where *Y*_*i*_ is the spectrum value obtained for the genotype *i*, *G*_*i*_ corresponds to the random genetic effect for genotype *i*, following a normal distribution $G\sim N(0,K{\sigma }_{G}^{2}$) with *K* the kinship (see above), and *e*_*ij*_ is the residual effect. We assumed that *G*_*i*_ and *e*_*i*_ were independent, identically distributed and followed a normal distribution.

Genomic heritability was then calculated as follows: ${H}_{\text{genomic}}^{2}=\frac{{\widehat{\sigma }}_{G}^{2}}{{\widehat{\sigma }}_{G}^{2}+{\widehat{\sigma }}_{e}^{2}}$, with ${\widehat{\sigma }}_{G}^{2}$ and ${\widehat{\sigma }}_{e}^{2}$, the REML estimates of *σ*^2^_G_ and *σ*^2^_e_ obtained using the R package sommer (Covarrubias-Pazaran 2016).

A spectral matrix *H* was calculated to represent the kinship between genotypes based on the NIR spectra similarity.

$$H= \frac{S{S}^{T}}{{N}_{\text{w}}}$$

With *S*, the raw NIR spectra matrix of dimension *N* × *N*_w_. The S matrix gathered the value of absorbance pretreated as presented above, for each genotype and each wavelength. Values were also centered and scaled. *N* represents the number of genotypes, and *N*_w_ represents the number of wavelengths. Mantel test using 999 permutations was realized to compare the spectral relationship matrix *H* with the genomic kinship matrix *K*.

GBLUP and HBLUP (using the *K* or *H* matrix, respectively) were used to predict seed germination-related traits which are defined as follows:

1 $$\text{GBLUP }{Y}_{i}= \mu +{G}_{i}+{\varepsilon }_{i}$$

2 $$\text{HBLUP }{Y}_{i}= \mu +{W}_{i}+{\varepsilon }_{i}$$

where Y_i_ is the mean phenotype value for the genotype *i*, *μ* is the intercept, *G*_*i*_ or *W*_*i*_ is the random genetic effect following a normal distribution $G\sim N(0,K{\sigma }_{G}^{2}$) with *K* the genomic kinship matrix (see above) or $W\sim N(0,H{\sigma }_{G}^{2}$) with *H* the hyperspectral matrix, and ε is the random residual effect following $\varepsilon \sim N(0,{\sigma }_{\varepsilon }^{2})$.

Both genomic (*K*) and spectral (*H*) matrices were included simultaneously in a GHBLUP model by integrating two variance–covariance matrices as follows:

3 $$\text{GHBLUP }{Y}_{i}=\mu +{G}_{i}+{W}_{i}+{\varepsilon }_{i,}$$

with $$G\sim N(0,K{\sigma }_{G}^{2})$$, and $$W\sim N(0,H{\sigma }_{\text{W}}^{2})$$.

GP and PP models were assessed for each trait using cross-validation (CV). Training set was composed of fourfold over five of genotypes chosen randomly, and the testing set was composed of the remaining fold. CV was repeated 100 times. These models were fitted using sommer R package (Covarrubias-Pazaran 2016). PA were estimated for each model by the Pearson correlation between the observed and the predicted values of the validation set.

By homology with the values estimated by genomic prediction, called GEBV, we called the values estimated by phenomic prediction PEPV, for phenomic estimated phenotypic values.

## Results

### Phenotypic distribution and correlations between traits

**Fig. 1:**
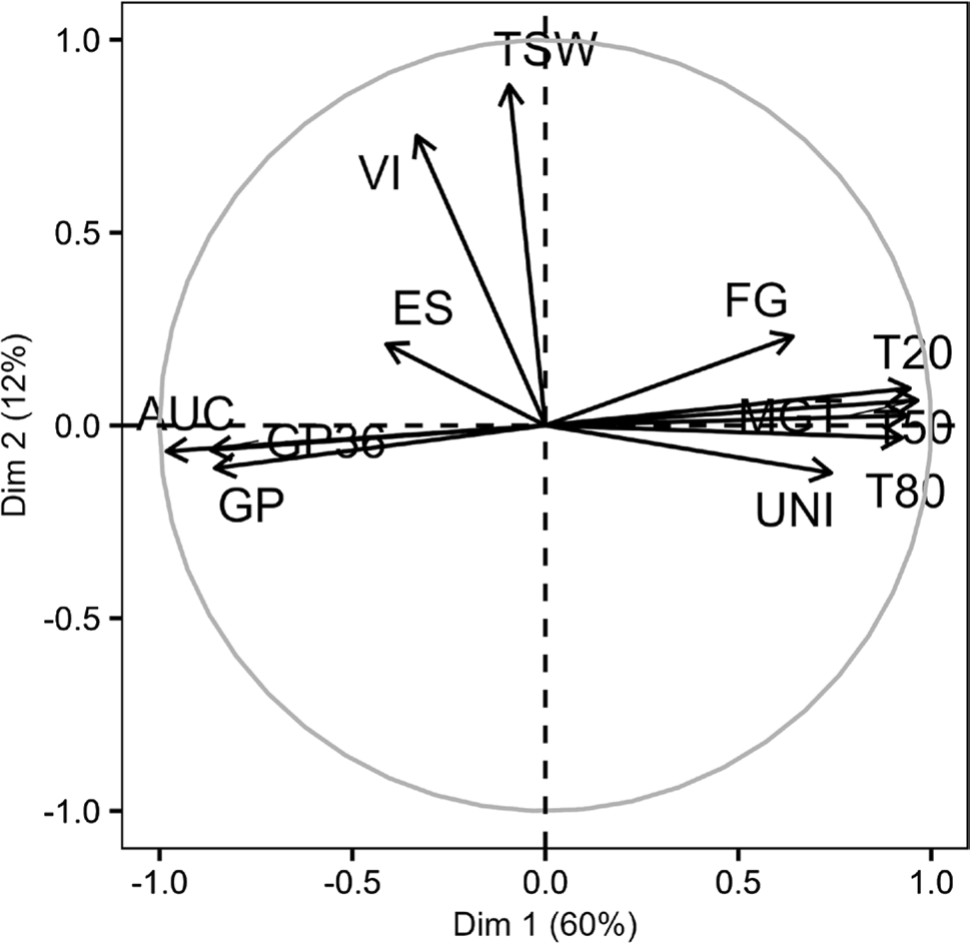
Plot of principal components for 12 variables associated with seed germination scored on 223 genotypes. Trait description is provided in Table 1

All germination-related traits displayed moderate-to-high heritabilities (Table 1). The highest heritabilities (> 0.60) were observed for GP36, T20, T50, T80, and MGT, and the lowest heritabilities were observed for VI (0.40) and UNI (0.43). Heritabilities could not be estimated for TSW and AUC as only one value per genotype was measured. GP, GP36, T20, T50, T80, AUC, and MGT were highly correlated as shown in Table 2. GP, AUC, and GP36 were positively correlated as well as T20, T50, T80, and MGT, which can be partially explained by the non-independence of traits. However, these two groups of variables presented negative correlations one with each other. VI and ES presented significant but moderate correlations with other traits (Table 2). TSW was only significantly correlated to UNI (−0.19), ES (0.19), and VI (0.52). These correlations were strengthened by the correlation circle of the PCA (Fig. 1). Indeed, AUC, GP, GP36, T80, T20, T50, and MGT were highly correlated to the first axis that gathered 60.42% of the variability while TSW and VI were correlated to the second axis that explained 12.43% of the variability. ES, UNI, and FG were less correlated to these two principal components.

**Table 2:** Correlation coefficients for trait pairs associated with seed germination scored on 223 rapeseed genotypes

	AUC	TSW	MGT	T20	T50	T80	UNI	GP	GP36	FG	VI
TSW	0.08										
MGT	−0.97***	**−**0.06									
T20	−0.94***	**−**0.04	0.96***								
T50	−0.96***	**−**0.05	0.96***	0.92***							
T80	−0.91***	**−**0.12	0.91***	0.84***	0.88***						
UNI	−0.73***	−0.19**	0.67***	0.53***	0.67***	0.88***					
GP	0.89***	0.10	−0.77***	−0.76***	−0.80***	−0.78***	−0.70***				
GP36	0.89***	0.07	−0.93***	−0.95***	−0.88***	−0.81***	−0.50***	0.68***			
FG	−0.61***	**−**0.08	0.6***	0.63***	0.55***	0.57***	0.40***	−0.55***	−0.62***		
VI	0.27***	0.52***	−0.29***	−0.29***	−0.27***	−0.23***	−0.18**	0.18**	0.28***	**−**0.11	
ES	0.35***	0.19*	−0.43***	−0.40***	−0.37***	−0.39***	−0.25***	0.20**	0.42***	−0.22**	0.14*

Significant values are shown in boldface

Significances were calculated at levels of: *p < 0.05, **p < 0.01, and ***p < 0.001

For details of trait abbreviations refer to Table 1 and “Material and methods”

### Germplasm structure using genomic, phenotypic, and spectral data revealed different patterns

**Fig. 2:**
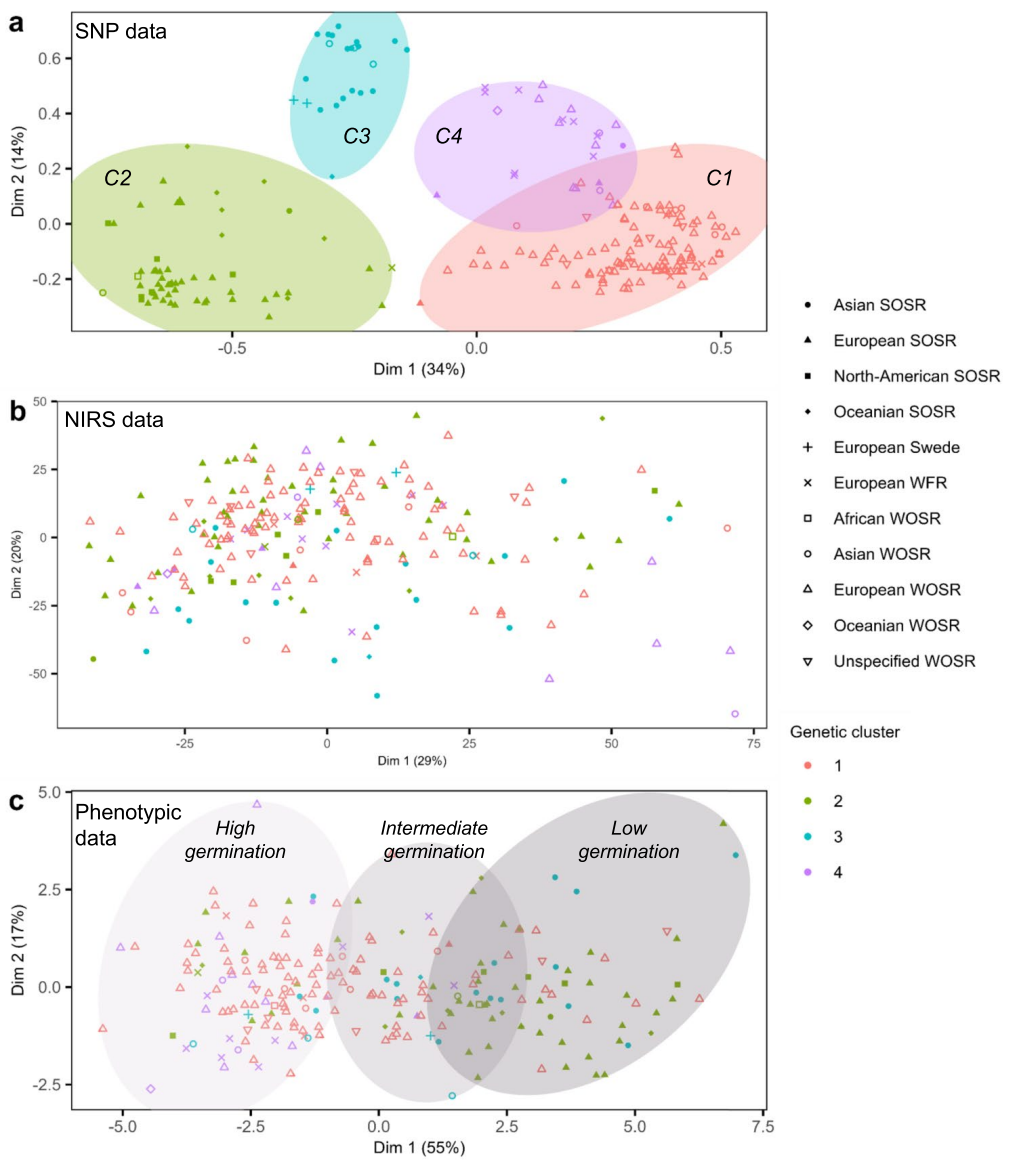
Genetic and phenotypic characterization of the panel used. Relatedness of 223 genotypes of rapeseed is highlighted by a principal coordinate analysis (PCoA) using the first two components using **a** SNP and **b** NIRS data acquired on seed samples. **c** PAM clustering on principal components of 12 variables associated with seed germination measured on 223 genotypes. Unfilled symbols correspond to winter types and plain symbols correspond to spring types

Genetic structure of the germplasm was assessed using the first two axes of the principal coordinates analysis using SNP (PCoA), explaining 48% of the genetic variance (Fig. 2a). The first axis that encompasses 34% of genetic variance clearly discriminated winter and spring types. The second axis (14% of the genetic variance) separated Asian genotypes on the one side and European and American genotypes on the other side. As a whole, four genetic clusters were identified using the PCoA clustering. Cluster 1 (*n* = 114, red) was mostly composed of European WOSR and WFR but also included some Asian WOSR. Cluster 2 (*n* = 62, green) was mostly composed of European and North American SOSR, but also gathered some Oceanian SOSR. Cluster 3 (*n* = 23, blue) corresponded to Asian SOSR and WOSR. Finally, cluster 4 (*n* = 24, purple) was composed of European winter fodder, Asian, and European WOSR. The mean nucleotide diversity *π* was computed on the whole germplasm and equaled 7.69e^−05^, indicating an important genetic diversity within the germplasm used in this study.

The relatedness between genotypes was also estimated using NIRS data (Fig. 2b). Heritabilities of the NIR spectra ranged between 0.00 and 0.95 regarding the wavelengths, with 34% and 16% of the wavelengths having a heritability superior or equal to 0.50 and 0.70, respectively (Online Resource 3). This indicates that NIRS is suitable for further genetic analysis. However, no cluster of genotypes was evidenced based on the NIRS data (Fig. 2b). In addition, when the genotypes were labeled according to their genetic cluster, no specific pattern was highlighted. Indeed, the correlation calculated using Mantel test between genomic (*K*) and hyperspectral (*H*) kinship matrices was equal to 0.051.

Based on the germination data, three phenotypic clusters of genotypes representing their seed germination performance were identified by PAM clustering (Fig. 2c). The first phenotypic cluster (from the left, light gray) corresponded to genotypes with a high germination capacity (high GP, GP36, AUC and low UNI, MGT, T80, T20, T50). The second phenotypic cluster (medium gray) consisted of genotypes with an intermediate behavior. Finally, the third cluster (dark gray) was composed of genotypes with poor germination ability (low GP, GP36, AUC and high MGT, T20, T50, T80, UNI). Each cluster gathered SOSR and WOSR types (Fig. 2c). Genomic clusters *C*1 and *C*4, which represented WOSR genotypes, were predominantly present in phenotypic clusters 1 (65% and 19%, respectively) and 2 (50 and 5%), i.e., clusters grouping genotypes with medium-to-high germination capacity. In contrast, genotypes from these genomic clusters (*C*1 and *C*4) were scarce in phenotypic cluster 3 (25 and 0%), respectively. Genotypes from clusters *C*2 and *C*3 were present at low frequency in the first phenotypic cluster (10 and 6%, respectively) and more frequent in the phenotypic clusters 2 (33 and 12%, respectively) and 3 (57 and 18%, respectively). Therefore, a link between the type or origin of rapeseed genotypes and their seed germination behavior was revealed.

### Identification of multiple QTL confirmed the polygenic determinism of seed germination

A total of 17 unique regions (QTL) were detected by GWAS for seed germination traits (GP36, MGT, T20, T50, and VI) and thousand seed weight (TSW), with an explained variance (*R*^2^) ranging from 6.30 to 8.78% (Table 3). Among these 17 QTL, three were identified for four germination traits (GP36, MGT, T20, and T50) on A06 (QTL.A06.1), A09 (QTL.A09.1), and C02 (QTL.C02.2). QTL.C01.1 was identified for GP36 and MGT, QTL.A06.2 for MGT and T50, and QTL.C04.2 for MGT and T20. Example of Manhattan and QQ plots for GP36 is available in Online Resource 4. Few QTL detected were trait-specific: three for GP36 (QTL.A07.1, QTL.C02.1, and QTL.C07.1), three for MGT (QTL.A10.1, QTL.C04.1, and QTL.C05.1), two for T50, (QTL.A07.2 and QTL.C07.2), one for TSW (QTL.A10.2), and twp for VI (QTL.C03.1 and QTL.C09.1) (Table 3). TSW QTL and VI QTL did not colocate with any QTL detected for the other traits. For most of the QTL, the favorable allele was the major allele in the germplasm (Table 3). Indeed, this was the case for one of the two QTL for VI, 5 QTL out of 9 for MGT, 3 QTL out of 4 for T20, and 5 QTL out of 6 for T50. On the contrary, for GP36, 5 QTL out of 7 had the minor allele as the favorable one. On the same line, the only QTL detected for TSW presented the minor allele as the favorable one. For all traits, a positive effect of the accumulation of favorable alleles was observed (Online Resource 5).

**Table 3:** QTL controlling germination-related traits detected in a population of 223 rapeseed through GWAS. QTL were ordered regarding their position on the genome. For each QTL, information is given about the peak and flanking markers, the peak SNP position, QTL size, as well as major and minor allele, followed by the minor allele frequency (MAF). For each trait, the SNP weight indicating the favorable allele version and the phenotypic variance explained by the SNP (R^2^) are represented. Positions are relative to the reference genome Darmor-*bzh* V10 (Rousseau-Gueutin et al. 2020)

QTL name	Chr	Trait	Peak SNP Peak position (pb)	Flanking marker 1/ Flanking marker 2	QTL size (kb)	Major/minor allele	MAF	SNP weight	R^2^
QTL.A06.1	A06	GP36	Bn_A06_p836912 (1,102,221)	Bn_A06_p709547/Bn_A06_p908271	279	T/C	0.15	**2.60**	7.60
		MGT						**−1.83**	8.46
		T20						**−1.81**	7.64
		T50						**−2.38**	8.78
QTL.A06.2	A06	MGT	Bn_A06_p14121814 (34,532,655)	Bn_A06_p19574863/Bn_A06_p18040423	4272	T/C	0.20	1.78	7.99
		T50						2.25	7.78
QTL.A07.1	A07	GP36	Bn_A07_p15220175 (21,453,055)	Bn_A07_p14390667/Bn_A07_p15885711	1530	A/G	0.25	**2.91**	8.59
QTL.A07.2	A07	T50	Bn_A07_p16553147 (22,869,578)	Bn_A07_p16157761/Bn_A07_p17026444	909	G/T	0.13	2.19	7.62
QTL.A09.1	A09	GP36	Bn_A09_p2733282 (4,101,875)	Bn_A09_p951202/Bn_A09_p7560188	8824	G/A	0.45	**−**4.00	8.31
		MGT						2.27	6.48
		T20						2.43	6.95
		T50						3.00	8.03
QTL.A10.1	A10	MGT	Bn_A10_p9459037 (13,661,179)	Bn_A10_p7835472/Bn_A10_p9645870	1990	C/T	0.27	**−**1.88	6.60
QTL.A10.2	A10	TSW	Bn_A10_p15838932 (18,901,950)	Bn_A10_p13817274/Bn_A10_p16933405	3226	G/T	0.28	**0.23**	7.44
QTL.C01.1	C01	GP36	Bn_scaff_15838_5_p446059 (3,600,398)	Bn_scaff_19244_1_p388798/Bn_scaff_17731_1_p308944	6736	T/G	0.45	**3.07**	7.47
		MGT						**−1.91**	6.73
QTL.C02.1	C02	GP36	Bn_scaff_18507_1_p957588 (35,173,323)	Bn_scaff_18507_1_p737065/Bn_scaff_17067_1_p669413	6469	A/G	0.13	**2.66**	6.99
QTL.C02.2	C02	GP36	Bn_scaff_16139_1_p720716 (61,523,315)	Bn_scaff_15918_1_p303294/Bn_scaff_16139_1_p41217	5361	T/C	0.32	**−**3.13	7.31
		MGT						2.15	8.30
		T20						2.16	7.75
		T50						2.47	7.04
QTL.C03.1	C03	VI	Bn_scaff_17440_1_p552871 (57,791,809)	Bn_scaff_19740_1_p203934/Bn_scaff_16394_1_p2229812	5070	T/C	0.39	**0.21**	6.50
QTL.C04.1	C04	MGT	Bn_scaff_18440_1_p77767 (9,746,347)	Bn_scaff_27469_1_p45054/ Bn_scaff_16576_1_p202308	12,852	T/C	0.24	2.53	6.69
QTL.C04.2	C04	MGT	Bn_scaff_24979_1_p13103 (53,828,390)	Bn_scaff_19208_1_p476848/Bn_scaff_20079_1_p289504	5714	T/C	0.36	1.88	7.05
		T20						1.93	6.73
QTL.C05.1	C05	MGT	Bn_scaff_16414_1_p1774629 (254,723)	Bn_scaff_23107_1_p7608/Bn_scaff_15712_10_p173981	5460	C/T	0.38	**−2.01**	6.30
QTL.C07.1	C07	GP36	Bn_A07_p3467400 (15,046,703)	Bn_A07_p3467400/Bn_scaff_16721_1_p1551224	28	C/G	0.07	3.01	8.69
QTL.C07.2	C07	T50	Bn_scaff_16110_1_p1076892 (54,582,081)	Bn_scaff_16110_1_p2439331/Bn_scaff_16110_1_p1076892	1509	A/G	0.07	2.22	7.91
**QTL.C09.1**	C09	VI	Bn_A09_p694190 (579,848)	Bn_scaff_16486_1_p88236/Bn_scaff_17297_1_p202207	6571	A/T	0.46	**−0.16**	6.84

SNP weight in boldface stands for minor alleles that are favorable

A search for candidate genes within the confidence intervals of the quantitative trait loci (QTL) identified two genes involved in germination (Online Resource 6): A10p30660.1_BnaDAR, located under QTL.A10.2, is an ortholog of *A. thaliana* gene involved in seed germination and radicle development, ARGINYL-t-RNA PROTEIN TRANSFERASE 1 (ATE1); and C03p76020.1_BnaDAR, located under QTL.C03.1, is an ortholog of another *A. thaliana* gene GDSL-motif lipase 1 (GDSL1).

### Relative performances of PP and GP at the whole panel level

**Fig. 3:**
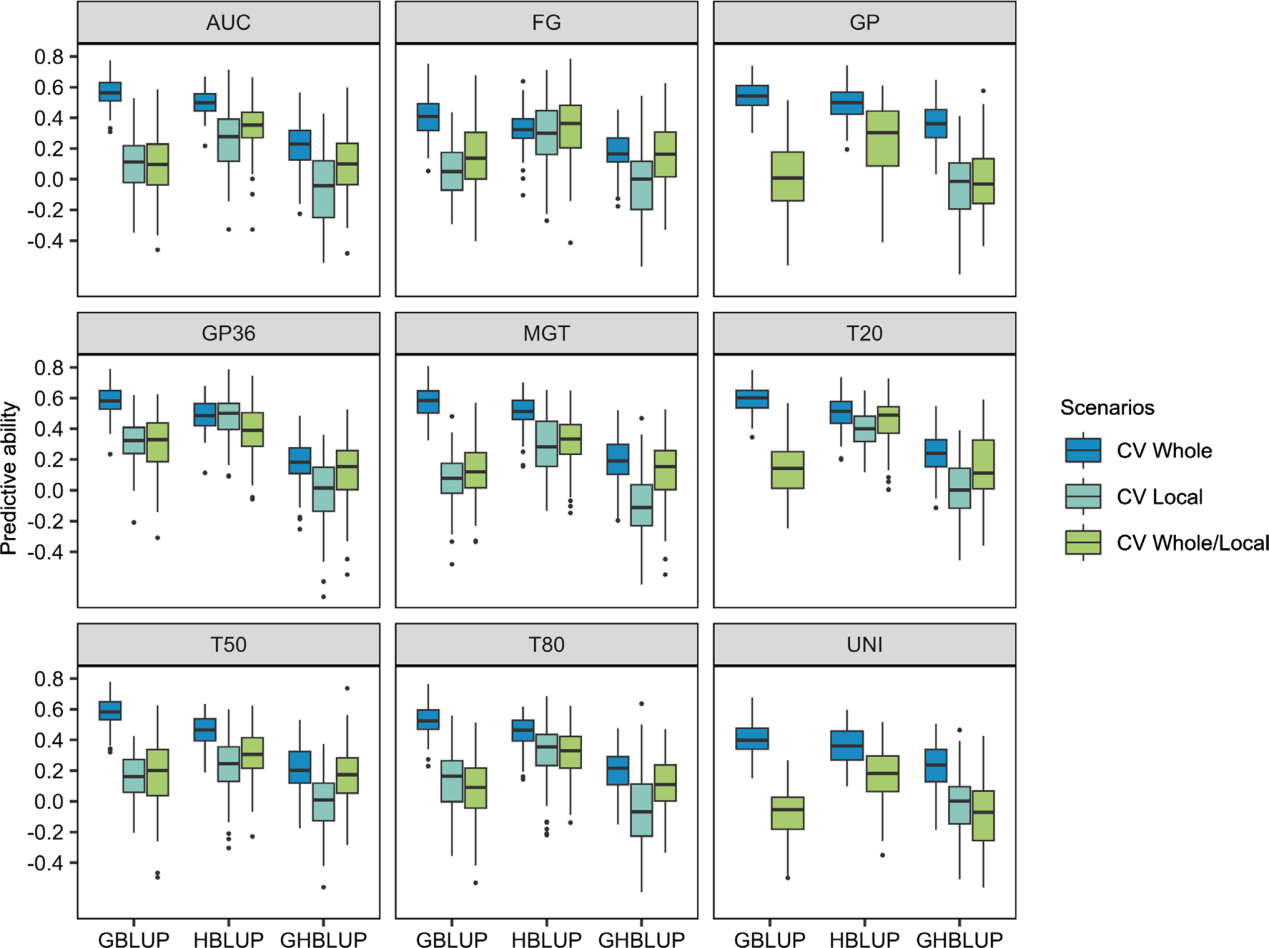
Predictive abilities of the nine seed germination-related traits using genomic (GBLUP), phenomic (HBLUP), or combined (GHBLUP) prediction models run for scenarios CV_whole_, CV_local_, and CV_whole/local_. Scenario CV_whole_ corresponds to the utilization of the whole germplasm for calibration and estimation of the PA, scenario CV_local_ corresponds to cluster 1 genotypes used for calibration and estimation of the PA. Scenario CV_whole/local_ corresponds to the utilization of the whole germplasm for calibration, and only genotypes of the cluster 1 were used for PA estimation. PA were obtained from a fivefold cross-validation with 100 repetitions. Each boxplot indicates the mean (bold line), the first and third quartiles (boxes), and the first and ninth deciles (whiskers) (color figure online)

**Fig. 4:**
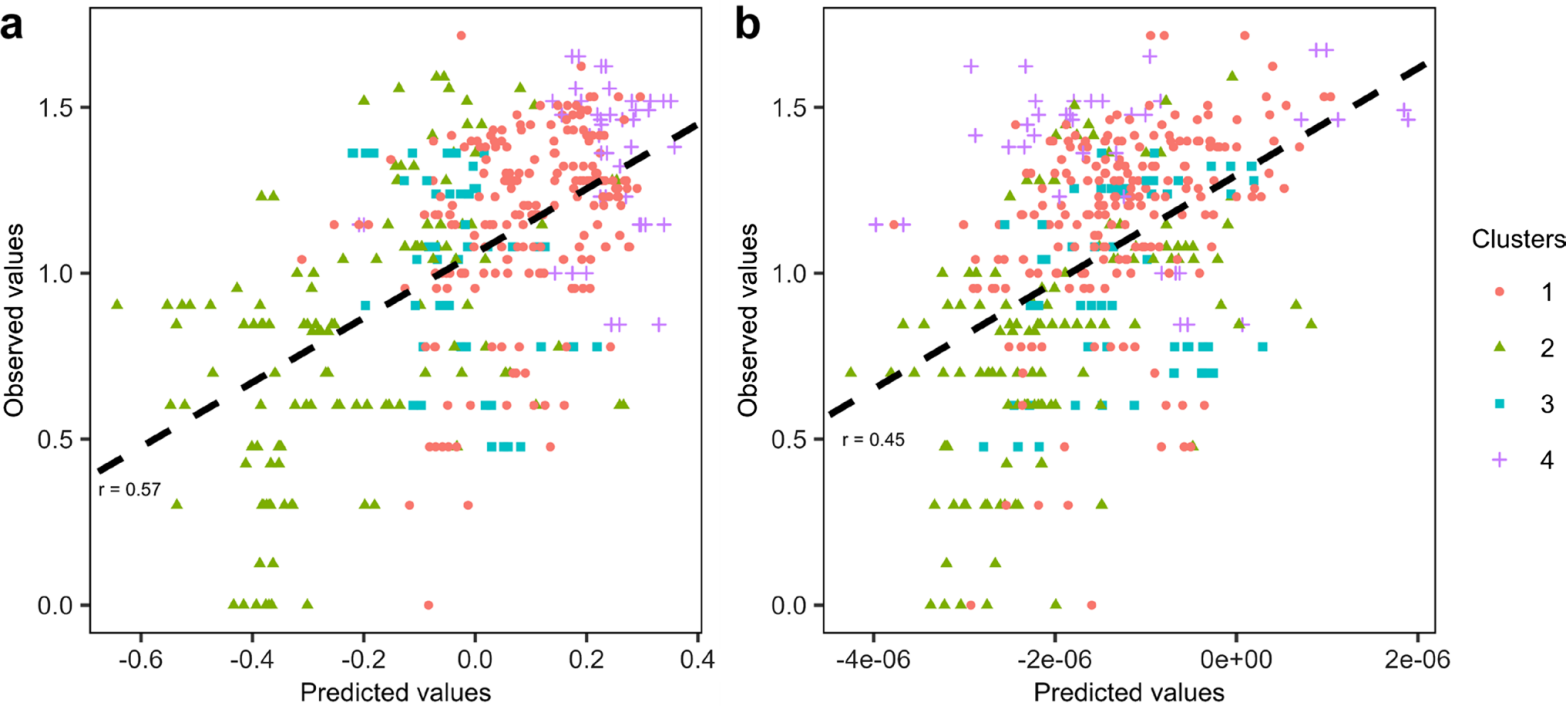
Predictive ability for GP36 using **a** genomic (GBLUP) and **b** phenomic (HBLUP) prediction models. Dots represent the observed and the predicted trait values across a fivefold cross-validation with 10 repetitions. Colors and dot types represent genomic clusters previously defined. The mean predictive ability (r) calculated across the entire dataset using a fivefold cross-validation with 100 repetitions is represented by a black line

Then, VI, ES, and TSW were dropped from the further analyses as they were weakly correlated to sensu stricto seed germination traits (Table 2). Predictions of seed germination traits were run either using genomic (GBLUP) or phenomic (HBLUP) prediction models (Fig. 3). Models that used a combination of the genomic and the spectral kinships were also tested (GHBLUP). In a first approach, we consider a first cross-validation (CV) scenario where the whole panel was used as training and prediction set (CV_whole_). More precisely, the training set included 80% of the whole population chosen randomly, the validation set being the remaining 20% of the whole population. Overall, low-to-medium predictive abilities (PA) were obtained, varying between 0.40 (FG, UNI) and 0.59 (T20) for GBLUP, 0.32 (FG) and 0.50 (T20, MGT) for HBLUP, and between 0.13 (GP36) and 0.34 (GP) for GHBLUP. For all traits, the PA of GBLUP appeared to be slightly higher or similar to the PA of HBLUP (Fig. 3). For instance, for GP36, the overall predictive ability of the genomic prediction was moderate (0.59), whereas the PA of HBLUP equaled 0.49. Furthermore, GHBLUP was less performant than GBLUP and HBLUP alone for all traits. Interestingly, when genotypes were labeled according to their genetic cluster affiliation, a separation between the different genetic clusters was revealed when looking at the GBLUP results, each genetic cluster corresponding to a specific stratum (Fig. 4). This result was observed for all traits (Online Resource 7). This highlighted the impact of the genetic structure on the genomic prediction model. Such pattern was less observed for the phenomic prediction model (Fig. 4, Online Resource 7). In conclusion, when the CV_whole_ scenario was considered, we found that PP was less performant but also less impacted by population structure than GP.

### Relative performances of PP and GP for unstructured population

As genetic structure influenced genomic predictions, particular attention was paid to investigating the most efficient way of predicting a specific genetic cluster. For this purpose, the genotypic cluster 1 was chosen as target population, as it is the only cluster that reached a sufficient size to allow model calibration (*n* = 114, but only 103 genotypes used due to missing NIRS, Fig. 2a). The cluster 1 gathered most of the winter oilseed rape genotypes. Therefore, two scenarios were compared (Fig. 3). In the first scenario, CV_local_, cluster 1 genotypes were used as calibration and validation sets. More precisely, 80% of cluster 1 genotypes were included in the training set, and the remaining 20% composed the validation set. In the second scenario, CV_whole/local_, 80% of the whole panel was used in the training set, while the validation set was composed of the genotypes of the cluster 1 that was not present in the calibration set. Each scenario was repeated 100 times. Some PA could not be calculated due to non-convergent model repetitions. A first observation was that PA calculated for these two scenarios were lower than PA obtained for the whole panel (Fig. 3), which can be explained by the fact that the genomic structure of the panel caused a bias in the PA estimation. Furthermore, PA of GBLUP or HBLUP models for scenarios CV_whole/local_ and CV_local_ were similar. But we observed that HBLUP model provided higher PA than the other two models, for all traits. In the context of the CV_local_ scenario, the results indicated that low-to-medium PA were obtained, with values ranging from 0.06 (FG) to 0.32 (GP36) for GBLUP and from 0.23 (T50) to 0.48 (GP36) for HBLUP. The highest difference in PA between the models was observed for FG (GBLUP PA = 0.09 and HBLUP PA = 0.25), while the lowest was observed for T50 (HBLUP PA = 0.23 and GBLUP PA = 0.15). In addition, GBLUP models outperformed GHBLUP models or obtained similar results (Fig. 3). Consequently, in case of unstructured population, PP led to higher PA values than GP.

### Choosing genotypes based on their PEPV rather than on their GEBV provided higher selection differentials

The performance of GBLUP and HBLUP models in selecting the top 10% of genotypes from the whole panel was investigated (Table 4). The mean of the BLUEs values of the whole panel for each trait was compared to the mean BLUEs value of different sets of genotypes. The first set corresponds to the top 10% of the whole panel based on the BLUEs values. The second set corresponds to the top 10% of the whole panel according to their GEBV. And the third set corresponds to the top 10% of the whole panel according to their phenomic estimated phenotypic values (PEPV). For each method, the mean BLUEs value was calculated. The selection differential (S) was estimated for each sampling method as the difference between the mean whole population BLUEs and the top 10% mean BLUEs. The selection differentials were higher when genotypes were chosen on their PEPV than when they were chosen according their GEBV (average increase in S between S estimated for GEBV and S for PEPV = 29%), indicating that selection decisions based on PEPV would be closer to phenotypic selection than GP-based selection. Concordances between best (and worst, respectively) (Online Resource 8) selected genotypes according to their BLUEs and their GEBV or concordances between best (and, respectively, worst) genotypes selected according to their BLUEs and their PEPV were studied using Jaccard’s similarity coefficient. This allowed to compare GP and PP ranking, for each scenario. Average concordances of 14.4% (BLUEs vs PEPV) and 7.2% (BLUEs vs GEBV) were obtained for the best 10% genotypes. Similar concordances were obtained for the 10% worst genotypes, with a concordance rate of 13.6% between BLUEs and PEPV, and 8.2% between BLUEs and GEBV.

**Table 4:** Average of the best linear unbiased estimates (BLUEs) for the whole germplasm, and the top 10% individuals selected based on their BLUEs, genomic estimated breeding values (GEBV), and phenomic estimated phenotypic values (PEPV), for each trait, using 210 rapeseed genotypes. For each average, the standard deviation is associated. The selection differential (S) was calculated for each values, as the difference between the germplasm mean and the mean of the top 10% genotypes

	Mean whole population	Top 10% BLUEs	S BLUEs	Top 10% GEBV	S GEBV	Top 10% PEPV	S PEPV
GP36	1.07 ± 0.36	1.55 ± 0.07	0.48	1.33 ± 0.21	0.26	1.33 ± 0.22	0.26
T50	51.85 ± 8.87	40.24 ± 1.77	**−**11.61	48.55 ± 4.83	**−**3.3	45.33 ± 5.06	**−**6.52
T20	41.23 ± 7.20	31.68 ± 1.30	**−**9.55	36.96 ± 4.57	**−**4.28	35.80 ± 4.51	**−**5.43
T80	63.48 ± 11.13	48.33 ± 1.97	**−**15.15	58.89 ± 6.56	**−**4.59	55.19 ± 5.60	**−**8.29
AUC	40.84 ± 11.79	60.14 ± 2.43	19.3	53.08 ± 5.50	12.24	53.55 ± 7.71	12.71
FG	30.16 ± 4.75	24.29 ± 0.85	**−**5.87	27.95 ± 3.27	**−**2.21	27.26 ± 2.39	**−**2.90
GP	2.53 ± 1.16	4.40 ± 0.33	1.87	2.81 ± 0.74	0.28	3.17 ± 0.84	0.64
MGT	52.22 ± 6.87	42.43 ± 1.62	**−**9.79	49.21 ± 4.97	**−**3.01	47.12 ± 4.03	**−**5.10
UNI	24.02 ± 7.50	14.49 ± 1.16	**−**9.53	20.83 ± 4.03	**−**3.19	20.03 ± 4.20	**−**3.99

## Discussion

In an attempt to get insights into the genetic architecture of seed germination in oilseed rape, we used a combination of genetic methods based on molecular as well as phenotypic and spectral predictors. The main results led to the highlight of 17 genomic regions that control seed germination-related traits in the large genetic diversity used. Moreover, genomic and phenomic prediction methods provided moderate-to-high predictive abilities, demonstrating the capacity to capture small additive and non-additive effects for seed germination. This study also provided the first application of phenomic prediction in oilseed rape and demonstrated the higher ability of phenomic prediction to estimate phenotypic values closer to BLUEs compared to genomic prediction.

### Alleles promoting seed germination are almost all fixed in oilseed rape

A total of 17 QTL with small effects were detected confirming the polygenic nature of seed germination as illustrated in the previous studies (Bettey et al. 2000; Basnet et al. 2015; Hatzig et al. 2015; Nguyen et al. 2018). Six of these genomic regions controlled several traits (e.g., GP36, MGT, T20, and T50), which was consistent with the high correlations between these traits (*r* < −0.88 or *r* > 0.96). This suggests pleiotropy or linkage drag. Therefore, it would be interesting to study the underlying genes of these six specific regions. We found a QTL in common with Hatzig et al. (2015) through the identification of a gene orthologous to *A. thaliana* ATE1, also characterized by Holman et al. (2009) as involved in seed germination. In addition, we found C03p76020.1_BnaDAR gene, highlighted by Ding et al. (2019) as being involved in seed germination in *A. thaliana* and *B. napus*. However, we found no other QTL in common between those identified in our study and those identified in the studies deciphering the genetic control of seed germination *sensus stricto* in *Brassica napus* (Hatzig et al. 2015; Nguyen et al. 2018; Boter et al. 2019; Gad et al. 2021; Luo et al. 2021), *Brassica oleracea* (Bettey et al. 2000), or *Brassica rapa* (Basnet et al. 2015) under optimal or stressed conditions. This lack of correspondence could be explained by the fact that these different studies (except Hatzig et al. (2015)) used restricted genetic diversity populations, limited either to WOSR or SOSR genotypes. Whereas we analyzed a larger genetic diversity.

To estimate the ability to improve seed germination in breeding by marker-assisted selection, we looked at the effect of QTL staking for each trait. The accumulation of several (> 3) favorable alleles increased seed germination speed. However, for most of the traits used in this study, the favorable alleles were also the most frequent alleles in our germplasm. Consequently, a low genetic gain is expected. Similarly, Hatzig et al. (2015) showed an improvement of T50 when stacking multiple favorable haplotypes. They also showed that the favorable haplotypes for this trait were the most frequent in the population, suggesting a strong selection for T50. Interestingly, some minor alleles were identified as favorable, for some traits involved in seed germination capacity, such as GP36. Therefore, improving seed germination capacity could still be achieved by targeting these favorable alleles presenting a low frequency in the population.

### SNP-based genomic structure of oilseed rape diversity did not match to the structure observed using spectral data, but influenced seed germination capacity

No difference between WOSR and SOSR was observed using NIRS data. Furthermore, kinship estimated based on NIRS data (*H* matrix) was not correlated to the one based on SNP data (*K* matrix). Similar results were obtained for soybean RIL populations (Zhu et al. 2021) or for Dent and Flint maize populations (Weiß et al. 2022). Brault et al. (2022) also reported a low correlation between NIRS-based *H* matrix and SNP-based *K* matrix using a diversity panel of grapevine. A single study for triticale revealed a *H* matrix well correlated to the genomic (Zhu et al. 2022). The low correlations between *H* and *K* matrices might result from different histories of domestication and selection or from different ranges of considered genetic diversity.

As shown in the previous studies of oilseed rape genetic diversity (Diers and Osborn 1994; Hasan et al. 2006; Bus et al. 2011), we observed two specific genomic clusters for winter and spring accessions using SNP data. Clustering on seed germination traits revealed a difference in performance between WOSR (*C*1 and *C*4) and SOSR (*C*2 and *C*3) genomic clusters. Nevertheless, within the panel, WOSR are represented in a similar proportion for each breeding period, unlike SOSR which were mainly bred between the 70 s and 80 s (Online Resource 1). These differences in breeding dates between WOSR and SOSR could partly explain the difference WOSR/SOSR observed for seed germination performance, thus confirming the hypothesis proposed when comparing H and K matrices. Indeed, Hatzig et al. (2018) showed that intensive selection conducted between the 70’s and 80’s to reduce erucic acid and glucosinolates content in seeds had a negative impact on seed germination capacity. Further investigation would be required to distangle the type effect (WOSR/SOSR) from the breeding history effect.

### Genomic and phenomic predictions allowed capturing weak polygenic effects

After seeking to highlight moderate additive effects by GWAS, genomic and phenomic prediction models were used to consider polygenic background. The combination of spectral and genomic data in the model called GHBLUP performed worse than HBLUP and worse or similar than GBLUP. Similarly, Brault et al. (2022), who identified weak correlations between K and H matrices, obtained no gain in PA by combining NIRS and SNP information. However, these results differed from the previous studies using GHBLUP model, which showed superior PA compared to GBLUP or HBLUP models on yield traits (Krause et al. 2019; Galán et al. 2020; Zhu et al. 2021; Robert et al. 2022). In the case of soybean, the rrBLUP model combining SNP and NIRS data showed a higher PA than the genomic rrBLUP, even if *K* and *H* matrices were not correlated (Zhu et al. 2021). The type of trait studied, its genetic architecture and, in particular, the proportion of phenotypic value explained by non-additive genetic effects, could explain these differences in GHBLUP performance. Therefore, this information could help to choose between genomic, phenomic, and combined predictions models. In particular, the use of NIRS data and PP models could be favored for traits moderately to strongly shaped by non-additive genetic effects. It is also necessary to remain cautious regarding the absorbance values for each wavelength. They can be influenced (i) by the environment in which the seeds were formed and stored, which can have an impact on the PA when predicting in independent environments, and (ii) by the environment in which the spectra were acquired (humidity and temperature), resulting in heterogeneous data over time. The decision to use some or all of the wavelengths, particularly with regard to their heritability, may also have an impact on the accuracy of phenomic predictions models. In addition, PA overestimation can result from the fact that the same samples are used both for the acquisition of NIRS data and for the acquisition of the phenotypic data. Dallinger et al. (2023) also warn against PA overestimation due to the correlation between traits to be predicted and seed compounds as well against unintentional selection of seed compounds in the selected population on the basis of its PEPV. Finally, it is necessary to remain vigilant regarding the traits chosen for prediction.

In this study, both GP and PP methodologies predicted well germination traits in the CV_whole_ scenario (up to 0.59 for GBLUP on GP36 and 0.50 for HBLUP on T20). However, we showed an impact of the genomic structure on the PA of GP but not with PP models (Fig. 3). This genomic structure impact on genomic prediction models was also reported previously (Riedelsheimer et al. 2013; Schopp et al. 2017; Werner et al. 2020). The absence of impact of genomic structure on PP is in accordance with Zhu et al. (2021, 2022) and Weiß et al. (2022) studies. Therefore, HBLUP models should be preferred to predict complex traits for genetically diverse germplasm.

### Interest of phenomic prediction in breeding to incorporate genetic diversity in elite pools

Our results confirm that seed germination performance could be increased through genomic and/or phenomic prediction, highlighting the interest in improving the genetic background. We showed that PP provided PEPV closer to BLUEs than GEBV. It would be necessary to confirm these results using independent phenotyping trials. According to the breeder equation (Lush 1943), the impact of PP could be interpreted according to the different terms of the equation: concerning the (i) the generation interval, no impact is expected as seed germination phenotype is acquired at the beginning of the cycle. Considering (ii) the intensity *i*, NIRS acquisition is simple, fast, and inexpensive, which would allow to screen a higher number of genotypes. Therefore, more resources could be allocated to construct the training population, which then could lead to a better PA and, therefore, to a better identification of the best promising individuals. This resources reallocation could subsequently also contribute to phenotype these promising individuals more reliably and in different environments. As a whole, PP is a mean to increase the selection intensity. However, a specific attention has to be paid to maintain genetic diversity within the breeding population to assure long-term genetic gain. This can be achieved, (i) by limiting or optimizing the selection intensity, (ii) by considering genetic diversity in the choice of parents to cross, using, for example, optimal contribution selection (Cowling et al. 2017), or (iii) by integrating exotic material into elite genotypes (Simmonds 1993). For this last point, we propose to use PP to efficiently identify genetic diversity to be valorized. Integrating this genetic diversity into elite germplasm would increase the frequency of favorable alleles in the population. Indeed, integrating exotic diversity into elite germplasm is one of the most important challenges in field crops (Cowling 2013), especially in oilseed rape. Oilseed rape has a narrow genetic diversity and could take advantage of this gain of diversity to overcome biotic and abiotic stresses while maintaining or increasing yields. For this purpose, the creation of pre-bridging and bridging populations (i.e., progeny obtain from elite x exotic crosses to be integrated into elite germplasm) have been proposed (Gorjanc et al. 2016; Allier et al. 2020; Sanchez et al. 2023). The construction of these (pre-)bridging populations could benefit from phenomic prediction. PP could help identifying material from a broad range of exotic resources for the creation and maintenance of pre-bridging and bridging populations.

### Supplementary Information

Below is the link to the electronic supplementary material.

Supplementary file1 (XLSX 3717 KB)

## Data Availability

The datasets generated and analyzed in this study are available using the following link during the review process (https://entrepot.recherche.data.gouv.fr/privateurl.xhtml?token=4050df7f-5caf-4683-8142-df8b870ef8d5) and will be freely available with a DOI if the article is accepted.

## References

[CR1] Abraham S, Golay MJE (1964). Smoothing and differentiation of data by simplified least squares procedures. Anal Chem.

[CR2] Albert E, Segura V, Gricourt J (2016). Association mapping reveals the genetic architecture of tomato response to water deficit: focus on major fruit quality traits. J Exp Bot.

[CR3] Albrecht T, Wimmer V, Auinger H-J (2011). Genome-based prediction of testcross values in maize. Theor Appl Genet.

[CR4] Allier A, Teyssèdre S, Lehermeier C (2020). Optimized breeding strategies to harness genetic resources with different performance levels. BMC Genomics.

[CR5] Araus JL, Kefauver SC, Zaman-Allah M (2018). Translating high-throughput phenotyping into genetic gain. Trends Plant Sci.

[CR6] Astle W, Balding DJ (2009). Population structure and cryptic relatedness in genetic association studies. Stat Sci.

[CR7] Basnet RK, Duwal A, Tiwari DN (2015). Quantitative trait locus analysis of seed germination and seedling vigor in brassica rapa reveals QTL hotspots and epistatic interactions. Front Plant Sci.

[CR8] Bettey M, Finch-Savage WE, King GJ, Lynn JR (2000). Quantitative genetic analysis of seed vigour and pre-emergence seedling growth traits in *Brassica*
*oleracea*. New Phytol.

[CR9] Boter M, Calleja-Cabrera J, Carrera-Castaño G (2019). An Integrative approach to analyze seed germination in *Brassica*
*napus*. Front Plant Sci.

[CR10] Boureau T (2020) PHENOTIC Platform

[CR11] Brault C, Lazerges J, Doligez A (2022). Interest of phenomic prediction as an alternative to genomic prediction in grapevine. Plant Methods.

[CR12] Browning BL, Zhou Y, Browning SR (2018). A One-penny imputed genome from next-generation reference panels. Am J Human Genet.

[CR13] Bus A, Körber N, Snowdon RJ, Stich B (2011). Patterns of molecular variation in a species-wide germplasm set of *Brassica*
*napus*. Theor Appl Genet.

[CR14] Clarke WE, Higgins EE, Plieske J (2016). A high-density SNP genotyping array for *Brassica*
*napus* and its ancestral diploid species based on optimised selection of single-locus markers in the allotetraploid genome. Theor Appl Genet.

[CR15] Covarrubias-Pazaran G (2016). Genome-assisted prediction of quantitative traits using the R Package sommer. PLoS ONE.

[CR16] Cowling WA (2013). Sustainable plant breeding. Plant Breed.

[CR17] Cowling WA, Li L, Siddique KHM (2017). Evolving gene banks: improving diverse populations of crop and exotic germplasm with optimal contribution selection. J Exp Bot.

[CR18] Dallinger HG, Löschenberger F, Bistrich H (2023). Predictor bias in genomic and phenomic selection. Theor Appl Genet.

[CR19] Danecek P, Auton A, Abecasis G (2011). The variant call format and VCFtools. Bioinformatics.

[CR20] Demilly D, Ducournau S, Wagner M-H, Gupta SD, Ibaraki Y (2014). Digital imaging of seed germination. Plant image analysis: fundamentals and applications.

[CR21] Diers BW, Osborn TC (1994). Genetic diversity of oilseed *Brassica*
*napus* germ plasm based on restriction fragment length polymorphisms. Theoret Appl Genetics.

[CR22] Ding L-N, Guo X-J, Li M (2019). Improving seed germination and oil contents by regulating the GDSL transcriptional level in *Brassica*
*napus*. Plant Cell Rep.

[CR23] Ducournau S, Feutry A, Plainchault P (2004). An image acquisition system for automated monitoring of the germination rate of sunflower seeds. Comput Electron Agric.

[CR24] Elliott RH, Mann LW, Johnson EN (2007). Vigor tests for evaluating establishment of canola under different growing conditions and tillage practices. Seed Technol.

[CR25] FAO (2023) FAOSTAT Food and agriculture data

[CR26] Finch-Savage WE, Bassel GW (2016). Seed vigour and crop establishment: extending performance beyond adaptation. J Exp Bot.

[CR27] Gad M, Chao H, Li H (2021). qtl mapping for seed germination response to drought stress in *Brassica*
*napus*. Front Plant Sci.

[CR28] Galán RJ, Bernal-Vasquez A-M, Jebsen C (2020). Integration of genotypic, hyperspectral, and phenotypic data to improve biomass yield prediction in hybrid rye. Theor Appl Genet.

[CR29] Gao X, Starmer J, Martin ER (2008). A multiple testing correction method for genetic association studies using correlated single nucleotide polymorphisms. Genet Epidemiol.

[CR30] Gao X, Becker LC, Becker DM (2010). Avoiding the high Bonferroni penalty in genome-wide association studies. Genet Epidemiol.

[CR31] Gorjanc G, Jenko J, Hearne SJ, Hickey JM (2016). Initiating maize pre-breeding programs using genomic selection to harness polygenic variation from landrace populations. BMC Genomics.

[CR32] Gower JC (1967). Multivariate analysis and multidimensional geometry. J R Stat Soc Ser D (stat).

[CR33] Haj Sghaier A, Tarnawa Á, Khaeim H (2022). The effects of temperature and water on the seed germination and seedling development of rapeseed (*Brassica*
*napus* L.). Plants.

[CR34] Hasan M, Seyis F, Badani AG (2006). Analysis of genetic diversity in the *Brassica*
*napus* L. gene pool using SSR markers. Genet Resour Crop Evol.

[CR35] Hatzig SV, Frisch M, Breuer F (2015). Genome-wide association mapping unravels the genetic control of seed germination and vigor in *Brassica*
*napus*. Front Plant Sci.

[CR36] Hatzig S, Breuer F, Nesi N (2018). Hidden effects of seed quality breeding on germination in oilseed rape (*Brassica*
*napus* L.). Front Plant Sci.

[CR37] Heffner EL, Jannink J-L, Sorrells ME (2011). Genomic selection accuracy using multifamily prediction models in a wheat breeding program. Plant Genome.

[CR38] Hickey JM, Chiurugwi T, Mackay I, Powell W (2017). Genomic prediction unifies animal and plant breeding programs to form platforms for biological discovery. Nat Genet.

[CR39] Holman TJ, Jones PD, Russell L (2009). The N-end rule pathway promotes seed germination and establishment through removal of ABA sensitivity in Arabidopsis. Proc Natl Acad Sci.

[CR40] Isik F, Bartholomé J, Farjat A (2016). Genomic selection in maritime pine. Plant Sci.

[CR41] Kaufman L, Rousseeuw P (1990). Partitioning around medoids (program PAM). Find Gr Data.

[CR42] Knoch D, Werner CR, Meyer RC (2021). Multi-omics-based prediction of hybrid performance in canola. Theor Appl Genet.

[CR43] Krause MR, González-Pérez L, Crossa J (2019). Hyperspectral reflectance-derived relationship matrices for genomic prediction of grain yield in wheat. G3 Genes Genomes Genetics..

[CR44] Lamichhane JR, Debaeke P, Steinberg C (2018). Abiotic and biotic factors affecting crop seed germination and seedling emergence: a conceptual framework. Plant Soil.

[CR45] Lane HM, Murray SC, Montesinos-López OA (2020). Phenomic selection and prediction of maize grain yield from near-infrared reflectance spectroscopy of kernels. Plant Phenom J.

[CR46] Lê S, Josse J, Husson F (2008). FactoMineR: An R package for multivariate analysis. J Stat Softw.

[CR48] Lippert C, Listgarten J, Liu Y (2011). FaST linear mixed models for genome-wide association studies. Nat Methods.

[CR49] Luo T, Zhang Y, Zhang C (2021). Genome-wide association mapping unravels the genetic control of seed vigor under low-temperature conditions in rapeseed (*Brassica*
*napus* L.). Plants.

[CR50] Lush JL (1943). Animal breeding plans.

[CR51] Mackay TFC, Stone EA, Ayroles JF (2009). The genetics of quantitative traits: challenges and prospects. Nat Rev Genet.

[CR52] Maechler M, Rousseeuw P, Struyf A, et al (2022) Cluster: cluster analysis basics and extensions

[CR53] Meuwissen THE, Hayes BJ, Goddard ME (2001). Prediction of total genetic value using genome-wide dense marker maps. Genetics.

[CR54] Muranty H, Troggio M, Sadok IB (2015). Accuracy and responses of genomic selection on key traits in apple breeding. Hortic Res.

[CR55] Nelson MN, Nesi N, Barrero JM (2022). Strategies to improve field establishment of canola: a review. Adv Agron.

[CR56] Nguyen TCT, Abrams SR, Friedt W, Snowdon RJ (2018). Quantitative trait locus analysis of seed germination, seedling vigour and seedling-regulated hormones in *Brassica*
*napus*. Plant Breed.

[CR57] Patti GJ, Yanes O, Siuzdak G (2012). Metabolomics: the apogee of the omics trilogy. Nat Rev Mol Cell Biol.

[CR58] Purcell S, Neale B, Todd-Brown K (2007). PLINK: a tool set for whole-genome association and population-based linkage analyses. Am J Hum Genet.

[CR59] Rajjou L, Duval M, Gallardo K (2012). Seed germination and vigor. Annu Rev Plant Biol.

[CR60] Resende MDV, Resende MFR, Sansaloni CP (2012). Genomic selection for growth and wood quality in Eucalyptus: capturing the missing heritability and accelerating breeding for complex traits in forest trees. New Phytol.

[CR61] Riedelsheimer C, Endelman JB, Stange M (2013). Genomic predictability of interconnected biparental maize populations. Genetics.

[CR62] Rincent R, Moreau L, Monod H (2014). Recovering power in association mapping panels with variable levels of linkage disequilibrium. Genetics.

[CR63] Rincent R, Charpentier J-P, Faivre-Rampant P (2018). Phenomic selection is a low-cost and high-throughput method based on indirect predictions: proof of concept on wheat and poplar. G3 Genes Genom Genet.

[CR64] Ritchie MD, Holzinger ER, Li R (2015). Methods of integrating data to uncover genotype–phenotype interactions. Nat Rev Genet.

[CR65] Robert P, Auzanneau J, Goudemand E (2022). Phenomic selection in wheat breeding: identification and optimisation of factors influencing prediction accuracy and comparison to genomic selection. Theor Appl Genet.

[CR66] Rooney TE, Sweeney DW, Sorrells ME (2022). Time series barley germination is predictable and associated with known seed dormancy loci. Crop Sci.

[CR67] Rousseau-Gueutin M, Belser C, Da Silva C (2020). Long-read assembly of the *Brassica*
*napus* reference genome Darmor-bzh. GigaScience.

[CR68] Sanchez D, Sadoun SB, Mary-Huard T (2023). Improving the use of plant genetic resources to sustain breeding programs’ efficiency. Proc Natl Acad Sci.

[CR69] Schopp P, Müller D, Technow F, Melchinger AE (2017). Accuracy of genomic prediction in synthetic populations depending on the number of parents, relatedness, and ancestral linkage disequilibrium. Genetics.

[CR70] Schrag TA, Westhues M, Schipprack W (2018). Beyond genomic prediction: combining different types of omics data can improve prediction of hybrid performance in maize. Genetics.

[CR47] Signal Developers (2014) Signal: signal processing. http://r-forge.r-project.org/projects/signal/

[CR71] Simmonds NW (1993). Introgression and incorporation. Strategies for the use of crop genetic resources. Biol Rev.

[CR72] Storey JD (2002). A direct approach to false discovery rates. J R Stat Soc: Ser B (stat Methodol).

[CR73] Voss-Fels KP, Cooper M, Hayes BJ (2019). Accelerating crop genetic gains with genomic selection. Theor Appl Genet.

[CR74] Wagner M-H, Demilly D, Ducournau S, Durr C (2011). Computer vision for monitoring seed germination from dry state to youg seedlings. Seed Test Int.

[CR75] Weiß TM, Zhu X, Leiser WL (2022). Unraveling the potential of phenomic selection within and among diverse breeding material of maize (Zea mays L.). Genes Genom Genet.

[CR76] Werner CR, Qian L, Voss-Fels KP (2018). Genome-wide regression models considering general and specific combining ability predict hybrid performance in oilseed rape with similar accuracy regardless of trait architecture. Theor Appl Genet.

[CR77] Werner CR, Gaynor RC, Gorjanc G (2020). How Population structure impacts genomic selection accuracy in cross-validation: implications for practical breeding. Front Plant Sci.

[CR78] Westhues M, Schrag TA, Heuer C (2017). Omics-based hybrid prediction in maize. Theor Appl Genet.

[CR79] Würschum T, Abel S, Zhao Y (2014). Potential of genomic selection in rapeseed (*Brassica*
*napus* L.) breeding. Plant Breed.

[CR80] Yu J, Pressoir G, Briggs WH (2006). A unified mixed-model method for association mapping that accounts for multiple levels of relatedness. Nat Genet.

[CR81] Zhu X, Leiser WL, Hahn V, Würschum T (2021). Phenomic selection is competitive with genomic selection for breeding of complex traits. Plant Phenom J.

[CR82] Zhu X, Maurer HP, Jenz M (2022). The performance of phenomic selection depends on the genetic architecture of the target trait. Theor Appl Genet.

